# Chronic chorioretinal detachment under normal intraocular pressure in eye with uveitic glaucoma after trabeculectomy: A case report

**DOI:** 10.1097/MD.0000000000018652

**Published:** 2020-01-10

**Authors:** Yutaka Sakurai, Kei Takayama, Tatsuhiro Abe, Masaru Takeuchi

**Affiliations:** Department of Ophthalmology, National Defense Medical College, Tokorozawa, Saitama, Japan.

**Keywords:** choroidal detachment, complication, glaucoma, surgery, uveitis

## Abstract

**Rationale:**

Choroidal detachment is a major postoperative complication of trabeculectomy. Postoperative choroidal detachment occurs with low intraocular pressure (IOP), and is naturally resolved by elevation of IOP. We report a case of chronic chorioretinal detachment (CRD) in the eye with uveitic glaucoma after trabeculectomy which persisted with normal IOP resistant for medication and required surgery.

**Patient concerns:**

A 63-year-old man was referred to our department with uncontrolled uveitic glaucoma in his right eye. At first presentation, IOP was 62 mm Hg in the right eye with opened angle, and active ocular inflammation was presented by moderate cell infiltration to the anterior chamber.

**Diagnosis:**

Uveitic glaucoma.

**Interventions:**

Trabeculectomy with mitomycin-C combined with phacoemulsification were performed without any surgical trouble. Postoperative inflammation in the anterior segment was mild, and IOP decreased to the middle-teen.

**Outcomes:**

At 19 days after surgery, the depth of the anterior chamber changed to shallow and CRD occurred in the inferior quadrant area. This complication could not be resolved by additional systemic corticosteroid medication and scleral fenestration. Although IOP was maintained in middle-teen range, suture fixation of the sclera flap and additional scleral fenestration were necessary to resolve CRD at 191 days after primary surgery.

**Lessons:**

In uveitic eye with uncontrolled ocular hypertension, severe CRD after trabeculectomy is able to occur even with normal IOP, which requires surgical procedure in addition to the medical treatment.

## 1 Introduction

Ocular hypertension (OH) is a common complication in any type of uveitis, which occurs at any time during the course of disease.^[1]^ Antiglaucoma agents, such as topical prostaglandin analogs, β-blockers, carbonic anhydrase inhibitors, and rho-kinase inhibitor are often used to reduce OH.^[2]^ However, when medication therapy is not sufficient, surgical procedure should be necessary, but the rates of postoperative complications in eyes with uveitis are higher than those without uveitis, and become more severe condition.^[3]^

Choroidal detachment is a major postoperative complication of trabeculectomy.^[4]^ In general, postoperative choroidal detachment occurs with low intraocular pressure (IOP) and is naturally resolved by elevation of IOP.^[5]^ Severe choroidal detachment is often accompanied with serous retinal detachment and is known as chorioretinal detachment (CRD). For CRD, systemic corticosteroid medication is used as the first line, and surgical procedures including scleral fenestration, or re-suturing of scleral flap, are performed if necessary. We report a case of chronic severe CRD persistent with normal IOP after trabeculectomy in the eye with uveitic glaucoma which needed scleral fenestration and re-suturing of scleral flap.

## 2 Case report

**Figure 1:**
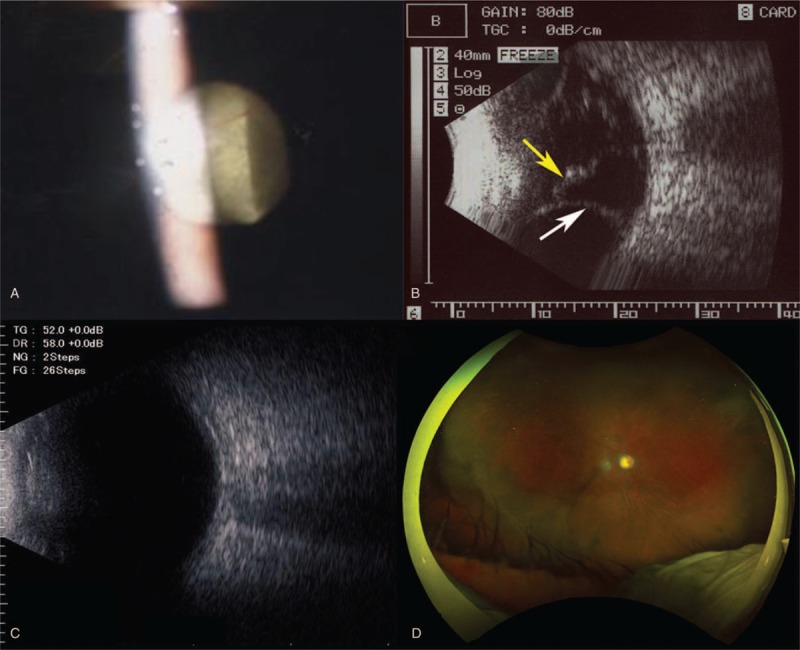
Microscopic examination, ultrasound examination, color fundus photo. Microscopic examination detected detached retina behind the lens (A). Ultrasound examination detected choroidal detachment (white allow) and serous retinal detachment (yellow allow) at postoperative 19 d (B) and these complications were resolved after additional treatment (C). Optic disc was changed to pallor (D).

**Figure 2:**
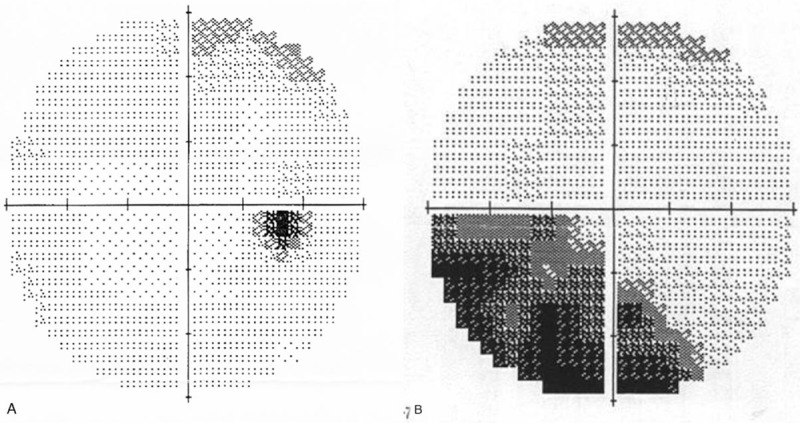
Changes of visual field. Visual field at first presentation (A) and at last visit (B). The visual field was measured by Humphrey Field Analyzer 30-2.

A 63-year-old man with uncontrolled uveitic glaucoma in the right eye was referred to our department. He had 10-years history of diabetes mellitus and 16-years history of anterior granulomatous uveitis and had been treated with topical antiglaucoma and corticosteroid agents, systemic corticosteroid medication (15 mg/day of prednisolone), and immunosuppressive agents. Systemic examination and polymerase chain reaction test in the aqueous humor could not detect the etiology of uveitis. At the first presentation, visual acuity and IOP were 20/20 and 62 mm Hg, and active ocular inflammation presented by moderate cell infiltration to the anterior chamber and peripheral anterior synechia of approximately 50% of total angle were observed in the right eye, Additional oral carbonic anhydrase inhibitor was initiated but IOP was still 47 mm Hg, and trabeculectomy with mitomycin-C (MMC) combined with phacoemulsification were performed without any surgical trouble. Postoperative 19 days, the depth of the anterior chamber with slight inflammation became shallow, and choroidal detachment occurred in the inferior quadrant area within middle-teen IOP (15 mm Hg) in the right eye. Additional systemic corticosteroid medication (40 mg/d of prednisolone) was initiated; however, choroidal detachment was worsened and developed to CRD (Fig. 1A and B ). From postoperative 120 days, scleral fenestrations were performed 3 times with monthly intervals, but the complications were not resolved. During the procedure, IOP was maintained in middle-teen range. At postoperative 191 days, re-suturing of the sclera flap and additional scleral fenestration were performed. IOP was elevated to 40 mm Hg and resolved these complications (Fig. 1C). Thereafter, IOP was reduced by hypotensive agents and subconjunctival needling and was maintained in high-teen range. Visual acuity was not changed during the course of treatment (20/20) and inflammation in the anterior segment was resolved by systemic immunosuppressive agents and topical/systemic corticosteroid, although optic disc changed to pallor (Fig. 1D) and visual field was defected (Fig. 2A and B).

## 3 Discussion

Surgical management of uveitis-associated glaucoma is often challenging and the rates of postoperative complications are high and the condition became severe, particularly in eyes with active inflammation.^[3]^ Multiple complicating factors are associated. Firstly, there is higher propensity for postoperative hypotony due to dysfunction of ciliary body impaired by chronic and relapsing ocular inflammation.^[6]^ Second, the use of antimetabolite such as MMC to inhibit the scarring responses causes further increase of the risk of hypotony.^[7]^ Third, postoperative inflammation is more accelerated in uveitic eyes following ocular surgery.^[8]^ For these reasons, previous studies have shown lower surgical success rates of trabeculectomy with antimetabolite in uveitic glaucoma compared with primary open angle glaucoma.^[9]^ In the present case, surgical procedure was performed in emergency because of severe OH resistant to anti-inflammatory medication. After trabeculectomy, although ocular inflammation was controlled by topical corticosteroid medication and systemic immunosuppressive agents, CRD occurred with normal ocular tension. Since additional systemic corticosteroid medication failed to resolve CRD, it is considered that noninflammatory factors associated with impaired production, outflow, and/or circulation of the aqueous humor would cause these complications.^[10]^ For treating chronic CD, systemic steroids do not appear to be effective, and surgical decompression of the vortex veins as they pass through the sclera has been described, but the most common treatment is full thickness sclerectomies to provide an exit for choroidal fluid.^[10]^ Therefore, scleral fenestration and elevating IOP by re-suturing were performed, although visual field was defected largely.

In the eye with uncontrolled uveitic glaucoma, severe CRD after trabeculectomy is able to occur even with normal IOP, which requires surgical procedures in addition to controlling inflammation.

## Author contributions

**Conceptualization:** Masaru Takeuchi.

**Data curation:** Yutaka Sakurai.

**Resources:** Tatsuhiro Abe.

**Supervision:** Masaru Takeuchi.

**Validation:** Kei Takayama.

**Writing – original draft:** Kei Takayama.

**Writing – review and editing:** Masaru Takeuchi.
